# Loss of the flagellum happened only once in the fungal lineage: phylogenetic structure of Kingdom Fungi inferred from RNA polymerase II subunit genes

**DOI:** 10.1186/1471-2148-6-74

**Published:** 2006-09-29

**Authors:** Yajuan J Liu, Matthew C Hodson, Benjamin D Hall

**Affiliations:** 1 Departments of Biology and Genome Sciences, University of Washington, Seattle, WA 98195, USA

## Abstract

**Background:**

At present, there is not a widely accepted consensus view regarding the phylogenetic structure of kingdom Fungi although two major phyla, Ascomycota and Basidiomycota, are clearly delineated. Regarding the lower fungi, Zygomycota and Chytridiomycota, a variety of proposals have been advanced. Microsporidia may or may not be fungi; the Glomales (vesicular-arbuscular mycorrhizal fungi) may or may not constitute a fifth fungal phylum, and the loss of the flagellum may have occurred either once or multiple times during fungal evolution. All of these issues are capable of being resolved by a molecular phylogenetic analysis which achieves strong statistical support for major branches. To date, no fungal phylogeny based upon molecular characters has satisfied this criterion.

**Results:**

Using the translated amino acid sequences of the RPB1 and RPB2 genes, we have inferred a fungal phylogeny that consists largely of well-supported monophyletic phyla. Our major results, each with significant statistical support, are: (1) Microsporidia are sister to kingdom Fungi and are not members of Zygomycota; that is, Microsporidia and fungi originated from a common ancestor. (2) Chytridiomycota, the only fungal phylum having a developmental stage with a flagellum, is paraphyletic and is the basal lineage. (3) Zygomycota is monophyletic based upon sampling of Trichomycetes, Zygomycetes, and Glomales. (4) Zygomycota, Basidiomycota, and Ascomycota form a monophyletic group separate from Chytridiomycota. (5) Basidiomycota and Ascomycota are monophyletic sister groups.

**Conclusion:**

In general, this paper highlights the evolutionary position and significance of the lower fungi (Zygomycota and Chytridiomycota). Our results suggest that loss of the flagellum happened only once during early stages of fungal evolution; consequently, the majority of fungi, unlike plants and animals, are nonflagellated. The phylogeny we infer from gene sequences is the first one that is congruent with the widely accepted morphology-based classification of Fungi. We find that, contrary to what has been published elsewhere, the four morphologically defined phyla (Ascomycota, Basidiomycota, Zygomycota and Chytridiomycota) do not overlap with one another. Microsporidia are not included within kingdom Fungi; rather they are a sister-group to the Fungi. Our study demonstrates the applicability of protein sequences from large, slowly-evolving genes to the derivation of well-resolved and highly supported phylogenies across long evolutionary distances.

## Background

The 9 + 2 flagellum is a major defining characteristic of eukaryotic organisms [1]. Of the three crown eukaryote taxa, only the fungi generally lack flagella, both in vegetative forms and sexual stages. Among lower fungi, however, flagellated gametes are found in a number of taxa. These organisms, coincidentally, are the fungi for which phylogenetic ascertainment is most problematic. This paper is addressed to the goal of obtaining a more robustly supported molecular phylogeny in order to determine whether a single loss event can have been responsible for the lack of flagella in the majority of fungi.

Fungi are one of the most ancient and diverse groups of eukaryotic organisms [2]. While their fossil remains extend back to 600 mya [3], molecular clock estimates place the origin of fungi at or before 1.5 bya [4]. The recognition, delimitation and typification of fungi have been formidable problems since the discovery of these organisms. The presence of chitin in the cell wall is a common character, but it is not unique to fungi [5]. Many fungi are saprobes; others are pathogens or mutualistic symbionts of plants and insects [5,6]. Despite this great variability in form and function, systematic studies based upon morphology and life cycle produced, by the mid 20^th ^century, a manageable number of major phyla. Ascomycota (yeasts and molds), Basidiomycota (mushrooms, smuts and rusts), and a number of taxa regarded as more ancestral forms constituted the group of organisms that were considered to be true fungi [5].

Molecular approaches to the study of eukaryote evolution began with the study of genes for small (5 S), then large (18S and 28 S) ribosomal RNA molecules. Major early contributions of rDNA studies to the circumscription of the monophyletic Kingdom Fungi showed that organisms such as Oomycetes (i.e. *Phytophthora *and *Achyla*) bore little similarity to the true fungi [7-11]. Subsequently, these organisms have been grouped with brown algae and other Stramenopila [12]. Conversely, rDNA studies were the first to identify *Pneumocystis carinii*, the infectious agent of AIDS-associated pneumonia, as a fungus belonging to the Ascomycota [13]. As fungal classification based on DNA sequences became a widely-used taxonomic tool, sequences of the ribosomal RNA molecules and ITS sequences were also shown to be useful for resolving very closely related fungal species from one another [14-16].

A profound understanding of the fungi and their interrelationships requires knowledge of the branching topology between major groups of fungi. Fungal phylogenies based on rDNA sequences are broadly in accord with morphology-based taxonomic concepts for Ascomycota and Basidiomycota, but are inconsistent with them for the earliest fungal lineages, namely the Zygomycota and Chytridiomycota [9,17-23]. With a few exceptions, species in Chytridiomycota have flagellated zoospores, appropriate to their aqueous native habitat, while Zygomycota, like Basidiomycota and Ascomycota, are without flagella. The phylogenetic studies of Zygomycota and Chytridiomycota based upon rDNA have repeatedly found that these two phyla were polyphyletic [20,24,25]. Specifically, *Basidiobolus ranarum *(Entomophthorales, Zygomycetes) consistently grouped with the Chytridiomycota and is most close to members of Neocallimasticales while other members of the Entomophthorales are closely related to *Allomyces *and other Blastocladiales [20,24,25]. A more recent study, based upon a combined 18S and 28S rDNA data set from fewer taxa, reached a similar conclusion [23]. In neither of these studies were the conclusions strongly supported by the data. The Glomales, a fourth important zygomycete taxon, came out as sister to the asco-basidiomycete lineage [20,23]. Other studies using rDNA have elevated the Glomales to the position of a fifth fungal phylum, Glomeromycota [23,26-28]. A literal interpretation of these and other rDNA studies might lead one to question the validity of the traditional subdivision of basal fungi into Zygomycota and Chytridiomycota. Conversely this raises several questions. Are there structural, physiological or genetic attributes of rRNA genes that compromise their usefulness for phylogenetic ascertainment at the ordinal level and above? If so, does the phylogenetic tree of fungal rRNA genes accurately reflect organismal relationships? The answers to these questions are essential to a general understanding of fungi, of their evolutionary history and of the degree to which vegetative and sexual stages in their life cycles are homologous. For example, if Chytridiomycota is indeed polyphyletic, this would mean that flagella were lost repeatedly during the evolutionary adaptation of fungi from water to land.

Although the most extensive broad phylogenetic studies of fungi have been done with rDNA, similar work with more limited sampling has used the mitochondrial genome or, alternatively, several nuclear protein coding genes, such as EF-1α, small portions of RPB1, and tubulin genes [9,19,20,23-25,29-31]. None of the studies with these protein coding genes sampled Glomales. Generally, molecular studies based on these genes show polyphyletic or paraphyletic Chytridiomycota because of the placement of the blastocladialean chytrids with the Zygomycota.[9,20,23-25,31]. A recent phylogenetic study showed that overall fungal phylogenies based on EF-1α sequences were poorly resolved [31].

To gain a more complete understanding of the relationships among fungal orders and phyla, we have used sequences of nuclear genes RPB1 and RPB2 to infer a phylogeny of these organisms. These genes encode the largest (210 kd.) and second-largest (140 kd) subunits of nuclear RNA Polymerase II. Because these genes have a functional role that is essentially general, transcribing all mRNA-encoding genes of the nucleus, their evolution is highly constrained and correspondingly slow. In studies of other fungal phyla [32,33], RPB1 and RPB2 made possible the ascertainment of deep phylogenetic branches with a high degree of confidence. The resolution and statistical support manifested in RPB1 and RPB2 phylogenies, as compared to that afforded by the best available alternative, 18S rDNA, is shown by a third analysis we present here. For the same taxa included in the RPB phylogenetic analysis, we have derived an 18S rDNA phylogeny for comparison.

The phylogenetic data across Kingdom Fungi for RPB1 and RPB2 has made it possible to examine another reported relationship that is surprising, that of the Microsporidia to fungi. On the basis of a phylogenetic analysis of α- and β-tubulin gene sequences, it has been proposed that Microsporidia are fungi and that they are a sister taxon to certain Zygomycetes[34,35]. This conclusion must be evaluated carefully because tubulins are uniquely susceptible to convergent changes. The macromolecular properties of tubulin that reflect their amino acid sequence likely play an important role in determining cell shape, a property that is highly variable among lower fungi. We chose, therefore, to examine the microsporidian RPB1 and RPB2 sequences in relation to those of many fungal taxa, to find out whether the position inferred [35] for Microsporidia within Kingdom Fungi could be confirmed.

## Results

### RPB1 and RPB2 sequences in fungi

**Figure 1:**
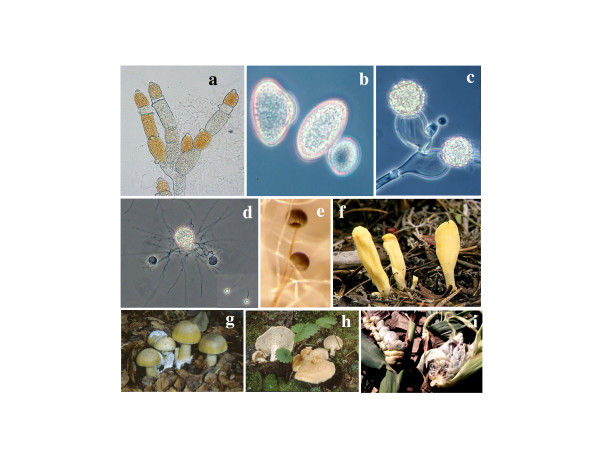
**Some of fungi used in this study**. a-d, Chytridiomycota, *Allomyces macrogynus *(a), *Coelomomyces stegomyiae *(b), *Monoblepharis sp. *(c), *Chytriomyces hyalinus *(d); e, *Rhizopus oryzae *(Zygomycota); f, *Neolecta vitellina (Ascomycota); *g-i, Basidiomycota,*Amanita phalloides *(g),*Hydnum repandum *(h),*Ustilago maydis *(i). Photographs are courtesy of Howard Whisler for b and c, Christopher Skory for e, Raymond Boyer for f, Steven Trudell for g and h, and Joe Ammirati for i.

**Figure 2:**
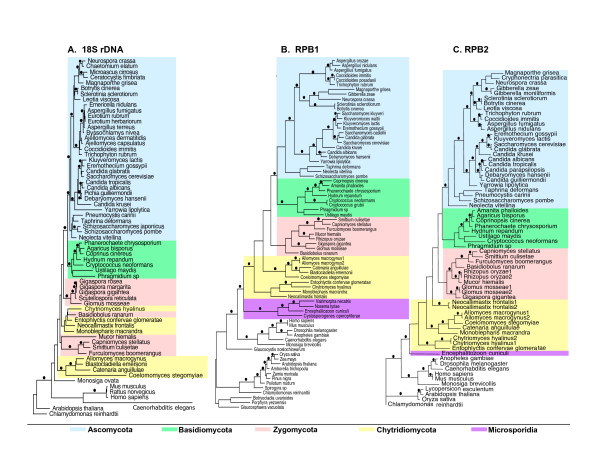
**Phylogenies of fungi based on 18S rDNA sequences (A), RPB1 (B) and RPB2 (C) protein sequences**. The phylogenies shown are the consensus trees of Bayesian Inference with maximum likelihood branch lengths evaluated using TREE-PUZZLE 5.2. The dots (•)above branches represent the braches with significant statistical support (>95% posterior probabilities of Bayesian inferences and >70% bootstrap values of parsimony analyses)

**Figure 3:**
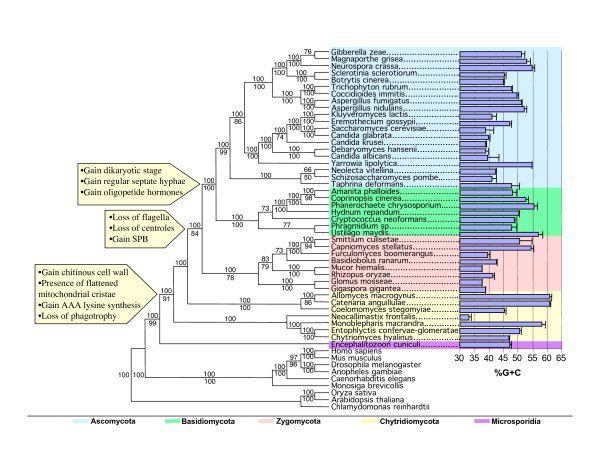
**The phylogeny based on the combined RPB1 and RPB2 protein sequences**. This phylogeny is obtained by Bayesian inference. Bayesian posterior probabilities (% are noted above individual branches and bootstrap values below the branches. The chart on the right side of the phylogeny depicts the %G+C of coding sequences of RPB1 and RPB2 with error bars for each taxon. The unique gains and losses of certain characters are mapped on the phylogeny.

**Figure 4:**
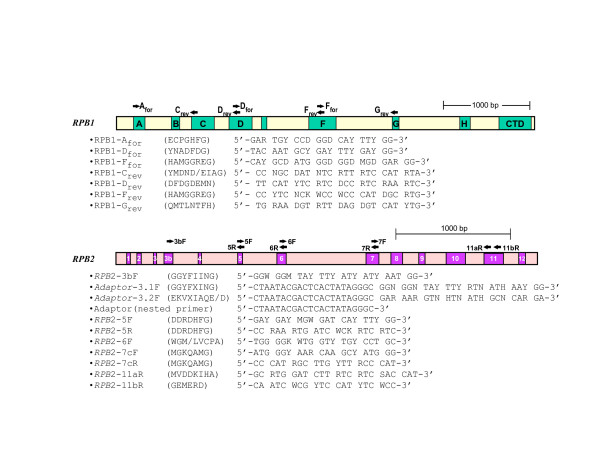
**RPB1 and RPB2 primers used in the study**. The long bars show the extent of the coding regions of RPB1 and RPB2, while the boxes with letters or numbers in them represent the amino acid motifs that are conserved throughout the eukaryotes. The arrows above show the positions of the primers used in this study, and their amino acid sequences and degenerate oligonucleotide sequences are listed below.

Taxa from four fungal phyla with diverse reproductive structures are sampled in this study (Figure 1). For each taxon, we sequenced at least 3.1 kb of RPB1 (A-G) and 2.7 kb of RPB2 (3–11) (Figure 4). When overlapping multiple PCR products were sequenced from individual taxa, there was no evidence for more than one copy of RPB1 or *RPB*2 in most of the fungal species examined. However, two RPB1 genes with slightly different sequences were found in *Allomyces macrogynus*, and two similar RPB2 copies were detected in *Glomus mosseae*, *Neocallimastix frontalis*, *Allomyces macrogynus *and *Chytriomyces hyalinus*. Two RPB2 sequences also were recovered by a search of the genomic sequence of *Rhizopus oryzae *[36]. For those species with paralogous gene copies, the percentage difference in nucleotide sequence ranged from 2.2 to 9.8, while the corresponding amino acid sequences had intraspecies variation of only 0.2 to 3.8%, since most of the nucleotide differences between them were synonymous substitutions. In both the *RPB1 *and *RPB2 *phylogenetic analyses, paralogs from the same species grouped closely together (Figure 2); therefore, only a single *RPB1 *and a single *RPB2 *gene were chosen for each of these six species in making up the combined data set (Figure 3).

Coding regions of the *RPB1 *and *RPB2 *genes of the major fungal taxa fall into a very distinct pattern as regards base composition. For three of the phyla, the range was similar, being from 39 to 55% G+C for Ascomycota, 48 to 57% for Basidiomycota and 37 to 54% for Zygomycota (Figure 3). In the Chytridiomycota the spread was much greater, ranging from a low of 33% G+C in *Neocallimastix frontalis *to a high of 66% in members of Blastocladiales (Figure 3). Previous studies have shown that phylogenetic analyses made by standard maximum likelihood or distance-based algorithms using nucleotide sequences give gene trees that differ significantly from one another when the GC content varies greatly between taxa [37]. For this and other reasons, we chose to use encoded protein sequences for phylogenetic analysis in this study.

### Phylogenetic relationships among major lineages of fungi based on RPB1 and RPB2 protein sequences

In phylogenetic analyses with the predicted amino acid sequences of RPB1 and RPB2, the majority of positions were informative based on the parsimony criterion (Table 1). The single most parsimonious tree obtained from RPB1, RPB2, or the combined dataset was in each case largely congruent with that from Bayesian analysis (Figures 2 and 3). The RPB1 and RPB2 phylogenies were also generally congruent (Figure 2), differing mainly as regards the branching within Taphrinomycotina and Chytridiomycota. In neither of these cases was this aspect of the topology highly supported (Figure 2).

**Table 1:** Summary of data sets of 18S rDNA, RPB1 and RPB2 and their branch support values under maximum parsimony (MP) criterion for single locus and combined analyses.

Characters	18S rDNA Nucleotide	RPB1 Amino acids	RPB2 Amino acids	RPB1 & RPB2 Amino acids
aligned	2388 bp	1119 (3357bp)	944 (2832bp)	2063 (6189bp)
excluded	649	58	43	101
included	1739	1061	901	1962
constant	745	257	244	501
parsimony-informative	738(31%)	723 (65%)	566 (60%)	1289 (63%)
No. of clades > 70% bootstrap	25 (59%)	31 (74%)	30 (71%)	33 (79%)

The phylogeny from parsimony analysis of the combined *RPB1 *and *RPB2 *dataset was highly resolved, as was that from Bayesian inference (Figure 3). Our criterion for statistically significant support of an individual node requires that the parsimony bootstrap value be >70% and the Bayesian posterior probability at least 0.95 [38]. Both methods of analysis found that monophyletic Ascomycota, Basidiomycota, and Zygomycota all were strongly supported (Figure 3). The most parsimonious tree and the tree from Bayesian inference were highly congruent; both trees included Glomales, Zygomycetes and Trichomycetes in the Zygomycota clade (Table 2, Figure 3). In the SH test, the phylogeny obtained by Bayesian analysis had the highest lnL score among the topologies tested, while for the most parsimonious tree lnL was lower, but not significantly so (Table 2, Figure 3). As regards the monophyly of Zygomycota, the less likely alternative hypothesis of Glomales being sister to Dikaryomycota and the Zygomycota being paraphyletic was not rejected (Table 2, Figure 3).

**Table 2:** Results of Shimodaira-Hasegawa (SH) tests for alternative hypotheses.

	SH test			
	
Hypothesis^a^	-lnL	Δ-lnL	*P*-value	Rejected ^b^
Bayesian phylogeny (= ML tree)	-78347.57	0	1.000	best
Microsporidia sister to Fungi (MP tree)	-78378.47	30.90	0.297	no
Microsporidia clustered with Blastocladiales	-78407.55	59.98	0.030	yes
Microsporidia clustered to other Members of Chytridiomycota	-78392.05	44.48	0.156	no
Microsporidia clustered with Zygomycota	-78437.65	90.08	0.002	yes
Microsporidia clustered with Dikaryomycota	-78453.52	105.95	0.002	yes
Microsporidia clustered with Animals	-78424.12	76.55	0.010	yes
Glomales sister to Dikaryomycota	-78383.99	36.42	0.248	no
Blastocladiales sister to zygo_trichomycetes	-78418.76	71.19	0.009	yes
Blastocladiales sister to Zygomycota	-78429.05	81.48	0.019	yes
monophyletic Chytridiomycota	-78376.30	28.73	0.367	no

Note: lnL, natural log likelihood; *P *values, the confidence limit for rejection of the alternative tree topology.

a. The maximum likelihood tree (ML tree), the most parsimonious tree (MP tree), and the most likely trees under the constrained conditions.

b. Best, the tree with the highest likelihood in comparison; Yes, rejected by maximum likelihood (*P *< 0.05); No, not rejected by maximum likelihood.

Ascomycota as a sister group to Basidiomycota had strong support (Figure 3). Basal to the clade of Ascomycota-Basidiomycota (Dikaryomycota) was monophyletic Zygomycota, a group with highly non-uniform morphology (Figure 3). Support for the node separating Chytridiomycota from the clade of Zygomycota-Dikaryomycota was remarkably strong (84% bootstrap, 100% Bayesian, Figure 3).

All phylogenies based upon RPB1 and RPB2 indicated that Chytridiomycota was paraphyletic and that it was the basal taxon of Kingdom Fungi (Figures 2 and 3). Within the Chytridiomycota were two well-supported lineages: Blastocladiales, which was sister to the Zygomycota-Dikaryomycota clade (Figure 3) and Chytridiales, the most basal clade of the fungi (Figure 3). *Monoblepharis *was sister either to the Blastocladiales clade in parsimony analysis or to the Chytridiales in Bayesian analysis (Figure 3), while *Neocallimastix *invariably occupied a position between the clades of Blastocladiales and Chytridiales. Although phylogenies based upon 18S rDNA sequences have found both Zygomycota and Chytridiomycota to be polyphyletic (Figure 2A), this alternative hypothesis is rejected in our SH test based on the combined data set of RPB genes (Table 2). Kingdom Fungi was strongly supported as a monophyletic group (Figure 3). Microsporidia were sister to the fungi in all phylogenies based upon RPB1 and RPB2 sequences (Figures 2 and 3). All alternative positions for the Microsporidia were rejected (Table 2), except for the possibility that Microsporidia cluster with the most basal members of Chytridiomycota (not including Blastocladiales).

### Nuclear 18S rDNA sequences in fungi

For each taxon sampled, we analyzed 1.8 kb (NS1-NS8) or more of aligned 18S rDNA. In the case of *Coelomomyces stegomyiae *the 18S rDNA is 2346 bp long and has many insertions. The 18S rDNA alignment contains regions of strongly conserved sequence interspersed with shorter regions prone to higher rates of substitution. Nuclear 18S rDNA exhibited a larger rate heterogeneity than do RPB1 and RPB2, as indicated by the higher proportion of invariable sites in the 18S rDNA (32% invariable sites, Table 1), and the relatively short interior branches and longer terminal branches in the 18S rDNA phylogeny (Figure 2A).

### Phylogenetic relationships among major lineages of fungi based on 18S rDNA sequences

The 18S rDNA phylogenies inferred by Bayesian or Parsimony analyses were closely similar (Figure 2A). Twenty-two most parsimonious trees were obtained in the Parsimony analyses. In the 18S rDNA phylogeny (Figure 2A), there was significant support for monophyly of both Ascomycota and Basidiomycota and for a sister relationship between them. These results were consistent with the RPB1 and RPB2 phylogenies. The main conflict between the 18S rDNA and RPB1 + RPB2 phylogenies concerned the relationships between members of Zygomycota and Chytridiomycota (Table 3). In the former, both Zygomycota and Chytridiomycota were polyphyletic, with members of Zygomycota falling into three clades. One of these, the Glomales, was sister to Dikaryomycota, another included *Mucor *of Zygomycetes and members of Trichomycetes, while in the third clade *Basidiobolus *of Zygomycetes was clustered with the members of Chytridiomycota (Figure 2A). Chytridiomycota was split between two clades, one being the Blastocladeales; the other including all other members of Chytridiomycota and *Basidiobolus *of Zygomycetes (Figure 2A). Neither of the above phylogenetic relationships in the 18S rDNA phylogeny was supported statistically (Figure 2A).

**Table 3:** Comparison between 18S rRNA and RPB gene phylogenies in the topology and statistical support for the major clades.

Major clade	18S rDNA	RPB1+RPB2
Monophyletic Ascomycota	100%, 75% ^a^	100%, 99%
Monophyletic Basidiomycota	100%, 89%	100%, 100%
Monophyletic Dikaryomycota	100%, 89%	100%, 100%
Monophyletic Zygomycota	No (polyphyletic)	100%, 78%
Clade of Asco-, Basidio- and Zygomycota	No ^b^	100%, 84%
Monophyletic Chytridiomycota	No (polyphyletic)	No (paraphyletic)
Monophyletic kingdom Fungi	72%, 71%	100%, 91%
Microsporidia sister to Fungi	No^c^	100%, 99%

a. The numbers are the values of Bayesian posterior probability and parsimony bootstrap support respectively.

b. Zygomycota did not form a clade with Ascomycota and Basidiomycota in the 18S rDNA phylogeny (Figure 2A).

c. Microsporidia is not included in the phylogenetic analyses based on 18S rDNA (Figure 2A) due to highly divergence of the sequences and difficulty in the alignment.

### Tests for congruency between 18S rRNA gene and RPB genes

Pairwise partition-homogeneity tests among three data sets indicated that 18S rDNA was neither congruent with the RPB1 nor the RPB2 data set, while the RPB1 and RPB2 data sets were congruent with one another.

We compared the major clades between phylogenies based on 18S rRNA and RPB genes and their respective bootstrap and Bayesian support values for the nodes subtending them (Table 3). In our RPB1 and RPB2 phylogenies, Chytridiomycota was shown to be the basal taxon in the Fungi and was separated from other fungi with high support (Figure 3, 100% Bayesian and 84% bootstrap). Therefore, the possibility that Zygomycetes were part of the earliest diverging fungal lineage as suggested by the 18S rDNA phylogeny was not considered further.

## Discussions and conclusion

The genes most extensively used for broad-scale fungal phylogeny are, in addition to RPB1 and RPB2: EF-1α, 5.8S, 18S, and 28S rRNA genes. Of these, the 18S rRNA gene most closely approaches RPB1 and RPB2 in its efficiency of providing phylogenetic resolution at this level. Consequently, we have included it in this study to make possible a direct comparison of the resolution afforded, to facilitate tests for congruency between the 18S rDNA and RPB trees and to search for any mutually well-supported conflicts between the two.

To effectively yield information about phylogeny places two requirements on the genes employed. First, there must be sufficient sequence variation across the taxa studied to resolve them. Second, the extent of variation must be low enough and broadly enough distributed so that multiple changes at the same site will be rare. These considerations imply that studies over long phylogenetic distances, such as those here that relate major fungal taxa, are best carried out using genes with slow but appreciable evolutionary rates.

The conflict among data sets, i.e. among rDNA, RPB genes and other protein-coding genes such as tubulins and EF-1α, can occur for several different root causes. First, the resolving power varies depending upon the data set. For example, both rDNA and EF-1α lack resolving power for internal branches in the tree due to lack of information and/or uncertainty in alignment [23,31]. Therefore, for many parts of the phylogenetic tree, the difference is between a node that is resolved and supported by the RPB data vs. a non-result (unresolved polytomy) or unsupported node for the rDNA/EF-1α data.

Second, the functional role of the gene can determine its utility in phylogenetics. Neutral or near-neutral mutations that occur over a long period of time in genes under purifying selection are the most reliable source of phylogenetic information. The difficulties inherent in genes undergoing adaptive evolution can seriously impact the deep branch topology in a broad scale phylogenetic study. For example tubulins, important elements in cell structures, can undergo adaptive evolution to different environmental challenges in the course of fungal evolution/speciation. They are not good candidates for phylogenetic study, especially for broad scale phylogeny. In contrast, RPB1 and RPB2 have provided the basic catalytic structure for RNA Polymerase II, an enzyme which has had the same relation to its DNA template and RNA product over the entire span of eukaryote evolution.

Although DNA sequences are helpful in resolving the topological relationships at the tips of the phylogeny, they are not as useful as protein sequences in determining the deeper branching topology in a study of this type. That is because, over the range of organisms we have studied, there is saturation of mutational changes at the 3^rd ^positions of codons, with resulting homoplasy if this information is included. If, to avoid that problem, only first and second codon positions are included, some information will be lost, since certain 3^rd ^position changes do result in amino acid changes. Analyzing the amino acid sequence with a JTT matrix is the best solution not only because it eliminates the noise at 3^rd ^positions but also because it takes into account the differences of selective pressure exerted by different amino acid substitutions.

### Chytridiomycota is paraphyletic and the basal taxon in fungi

Determining the phylogenetic relationship between Chytridiomycota and Zygomycota is a central issue in fungal evolutionary biology and a controversial one. It relates both to the question of which phylum is basal in Kingdom Fungi and to the mode of evolution of the flagellum, the lack of which differentiates most fungi from metazoans and flagellate protists. Members of Chytridiomycota are considered to be the basal lineage of fungi since, like metazoans, they use glycogen to store energy and have flagellated spores (zoospores) but, like other fungi, they have chitinous cell walls, flattened mitochondrial cristae, and use the AAA lysine synthesis pathway [39,40].

The RPB1 and RPB2 phylogenies show Chytridiomycota to be the basal taxon in the Fungi (Figures 2 and 3). Chytridiomycota is paraphyletic in the combined RPB1 and RPB2 phylogeny and consists of two major lineages; one being the Blastocladiales, the other including Chytridiales-Spizellomycetales-Monoblepharidales with Neocallimasticales as an outlier (Figure 3). These results are in accord with traditional morphological and ultrastructural studies of Chytridiomycota. A cladistic analysis of thallus morphology and ultrastructure showed three clades within the Chytridiomycota: Blastocladiales, Neocallimasticales, and Spizellomycetales-Chytridiales-Monoblepharidales [41]. A consideration of both the molecular and morphological evidence identifies the lineage containing the Monoblepharidales, Chytridiales and their close relatives as the most basal one in Kingdom Fungi.

### The transition from water to land happened only once during the evolution of fungi

Members of Chytridiomycota can exist in either unicellular or filamentous form. They are the only fungi that typically reproduce by forming flagellated zoospores as part of the life cycle. Chytridiomycota are considered to be aquatic because their unwalled and flagellated zoospores require water for dispersal although some of them have lost their flagella and produce amoebae instead of zoospores (i.e. *Amebochytrium*).

An assemblage containing all Ascomycota, Basidiomycota, and Zygomycota is a well-supported clade in these studies (Figure 3). All taxa within it lack flagella, contrasting with the Chytridiomycota which are mainly flagellated. Thus the flagellum was lost as a single event, coinciding with the loss of 9+2 microtubule centrioles, the appearance of the spindle pole body (SPB) as microtubule organizing center for mitotic and meiotic nuclear division and the shift from aqueous to terrestrial growth habit.

With the loss of motile cells, alternative methods of gamete release and dissemination evolved in fungi. Both mitotic and meiotic spores act to facilitate long-distance dispersal and resistance to adverse environmental conditions. Sporangiospores and zygospores formed internally were retained in most Zygomycota [42]. Novel mechanisms for conidium and meiospore formation and ballistosporic discharge have evolved in the Ascomycota and Basidiomycota.

### Zygomycota is monophyletic with significant statistical support

The unifying characteristics of Zygomycota are: mostly coenocytic hyphae (lacking regular septation), formation of highly resistant zygotes by the fusion of gametangia, and the absence of flagellated cells and centrioles [5]. The Zygomycota consist of two classes, the Trichomycetes and Zygomycetes[5]. All members of Trichomycetes are obligately associated with living arthropods [43]. Traditionally Zygomycetes consist of many orders including the Glomales, asexually reproducing soil fungi with very large spores which form endomycorrhizae with many vascular plants [27,44,45]. Previously published molecular phylogenies based on rDNA, β-tubulin, and EF-1α found Zygomycota to be non-monophyletic [9,20,21,23-25,27,29,34,46,47].

The phylogeny inferred in this paper from RPB1 and RPB2 sequence data supported a monophyletic Zygomycota that encompassed Zygomycetes, Trichomycetes, and Glomales (Table 3 and Figure 3). A shared derived trait uniting the Zygomycota is the production of asexual nonflagellate mitospores in sporangia (Figure 3). Our SH tests show that a monophyletic Zygomycota including Glomales has higher likelihood than a clade of Glomales with Ascomycota and Basidiomycota, however, the latter hypothesis is not rejected (Table 2).

### The relationship of Microsporidia to fungi

Microsporidia are widespread, obligate intracellular parasites of animals [48]. The view that Microsporidia belonged to an ancient group, the Archaezooans, that predated mitochondrial endosymbiosis was based upon the small size of their ribosomes and their lack of typical eukaryotic cytoplasmic organelles [49]. Early phylogenetic studies, using sequences of microsporidial 18S rDNA [50], EF-1α and EF-2 [51,52] also seemed consistent with a very early divergence of this taxon. Further analyses, however, attributed this conclusion to high rates of sequence divergence in microsporidial 18S rRNA and EF-1α gene sequences and consequent long branch attraction, leading to an incorrect assignment of phylogenetic position [53-55].

Relatedness of the Microsporidia to Fungi was suggested by subsequent phylogenetic studies using the genes RPB1, TBP, vacuolar ATPase subunit A, Valyl-tRNA synthetase, nuclear 28S rDNA, and two genes encoding tubulins[53,55-62]. Specialized features of the microsporidial RPB1 and EF-1α genes link microsporidia to the "crown" eukaryote group [53,63] and a relationship to fungi is also suggested by the presence of chitin in the spore during the reproduction of Microsporidia. Strong evidence against the Archaezooan hypothesis came from the finding, in the genome of *Encephalitozoon cuniculli*, of genes that clearly are of mitochondrial origin [58,59,62], implying that a secondary loss of mitochondria occurred during the evolution of Microsporidia.

While the preceding studies suggested a close relationship between Microsporidia and Fungi, they left open two possibilities: either that Microsporidia are a taxon within Kingdom Fungi or that Fungi and Microsporidia are sister to one another. These remain open because, in the initial study, no representatives were included from the two basal taxa of Fungi, the Chytridiomycota and Zygomycota [53,55-62]. A subsequent phylogenetic study, based on α- and β-tubulin sequences did include representatives of the basal fungal groups Zygomycota and Chytridiomycota. The phylogeny inferred from these data placed Microsporidia within Zygomycetes, in a position close to Entomophthorales and Zoopagales [34,35]. Because of the role that tubulin proteins play in determining cell shape, there is a strong possibility that in this instance, the affinity between Microsporidia and Zygomycetes results from common responses to developmental or environmental challenges and not from common descent. Between different groups of organisms, the rate of evolutionary change of tubulin genes is highly variable. For example, in members of Chytridiomycota, tubulin gene sequences are relatively conserved, while both in Zygomycetes and Microsporidia these genes diverged rapidly [34,35,55]. A likely consequence of this rate difference is long-branch attraction between Zygomycete and Microsporidial sequences.

It has been proposed by Cavalier-Smith [48] that Microsporidia evolved from harpellalean fungi (Trichomycetes). Three taxa of Harpellales (Trichomycetes) and other Zygomycota are included in this study; SH tests rejected the hypothesis of a harpellalean or zygomycete origin of Microsporidia (Table 2).

The results we have obtained show definitively that Microsporidia occupy a phylogenetic position outside Kingdom Fungi. Phylogenies inferred from RPB1, RPB2, and their combined sequences all show that Microsporidia are the sister taxon to fungi (Figures 2 and 3). In contrast to all of the other phylogenetic associations previously suggested for Microsporidia [34,35,64], this one receives excellent statistical support from the data. The relevant node has 100% Bayesian posterior probability and 99% bootstrap support in Figure 3. Statistical tests of various alternative derivations of Microsporidia (SH test, Table 2) rejected not only the hypothesis of an origin of Microsporidia within Zygomycota, but also rejected a close relationship either to Dikaryomycota or to Metazoa.

Our results suggest that the loss of flagella and their 9+2 microtubule structure in the members of Microsporidia is a separate event from the loss event that led to Zygomycota, Basidiomycota and Ascomycota. We envision the common ancestor of Fungi, Microsporidia and Metazoans as a unicellular, flagellated heterotroph, based upon comparisons between basal fungi, Microsporidia, basal Metazoans and associated groups such as Choanoflagellates. Our results suggest that loss of the flagellum happened at only one point in the evolution of fungi enabling the derived fungal phyla to adapt from the aquatic environment to a terrestrial one.

## Methods

### Materials

Fifty-eight taxa were used, including four Microsporidia, nine members of Chytridiomycota, eight zygomycetes, nine basidiomycetes, and thirty-two ascomycetes. As outgroups, ten taxa of animals, plants and protists were included in the analyses. We sampled four of the five orders of the Chytridiomycota as well as four orders representing all the classes of Zygomycota [5]. The sources of fungal strains and GenBank accession numbers for nuclear 18S rDNA, *RPB*1 and *RPB*2 gene sequences are listed in Supplementary Table [see Additional file 1]. The diverse reproductive structures of some representatives from the major fungal lineages are shown in Figure 1.

### Molecular techniques and phylogenetic analyses

The methods for fungal culture, DNA isolation, PCR amplification, cloning, and DNA sequencing have been described [65]. Primers NS1 through NS8 were used in this study to amplify nuclear 18S rDNA [66]. The set of general oligonucleotide primers used in this study for amplifying regions A to G of RPB1 and regions 3 to 11 of *RPB*2 genes are listed in Figure 4. All pairs of primers were used in the initial PCR amplification. For RPB1, Dfor and Frev usually work well for most fungi. Species specific primers were designed in the D-F region and paired with Afor and Grev for subsequent amplifications for some fungi (Figure 4). For RPB2, regions 5–7 and 7-11a were usually amplified easily by PCR. Species specific primers were designed in the region of 5–7 and paired with 3bF, 3.1F-adaptor, 3.2F-adaptor and 11aR for subsequent amplifications for some fungi (Figure 4). For certain fungi, nested PCR with the primer of adaptor sequence and species-specific primer were performed to amplify the 3–5 region of *RPB*2.

The nucleotide sequences of nuclear 18S rDNA and the amino acid sequences of RPB1 and RPB2 translated from DNA sequences were aligned using Clustal X [67], with subsequent visual adjustment, resulting in 2388, 1119 and 944 aligned positions, respectively, including gaps. The regions that could not be aligned reliably were removed leaving a total of 1739 positions for 18S rDNA, 1061 positions for RPB1 and 901 positions for RPB2 to use in phylogenetic analyses. Bayesian inference and maximum parsimony phylogenetic analyses were carried out for 18S rDNA, RPB1, and RPB2 singly, and for the combined protein sequences. To detect topological incongruence among data partitions of 18S rDNA, RPB1 and RPB2, pairwise partition-homogeneity tests among three data sets were conducted using PAUP* 4.0b10.[68] with parsimony criterion and heuristic search (10,000 replications).

The model selection approach, the Akaike Information Criterion (AIC), was used to estimate the best-fit model for Bayesian methods using MODELTEST 3.7[69,70]. A general time reversible (GTR) model including a proportion of invariant sites and a gamma distribution parameter was selected as the best-fit model for the 18S rDNA data set. For each dataset, Bayesian phylogenetic analyses with Markov chain Monte Carlo (MCMC) sampling was conducted using MrBayes v3.1 [71]. Six independent MCMC runs were carried out using the GTR model for the nucleotide substitution and JTT model for amino acid substitution. In addition, the proportion of invariable sites and a gamma distribution parameter to allow for rate heterogeneity among sites (six categories) and uniform prior probabilities and tree topologies were implemented in the analyses. The six runs included one run with 3 × 10^6 ^generations, two runs with 2 × 10^6 ^generations and three runs with 1 × 10^6 ^generations to ensure a sufficient number of generations and sampling of the same posterior probability landscape. Each run started with random trees for each of four simultaneous chains, resulted in concordant joint posterior probability distributions for the topology. The sampling was done every 100^th ^generation for each run. The samples before the convergence of the Markov chain were discarded for each run. The remaining samples from each run were combined into a single file with a total of 97830, 98095, 98876, and 98970 phylogenetic trees for the data sets of 18S rDNA, RPB1, RPB2 and combined RPB1 and RPB2, respectively. These were then imported into PAUP* 4.0b10 to compute the 50% majority rule consensus tree. The percentages for the branches in the consensus tree represent the Bayesian posterior probabilities which are the rough equivalent of a maximum likelihood search with bootstrapping[71]. The resulting consensus tree from Bayesian analyses for each dataset was evaluated for branch lengths using TREE-PUZZLE 5.0 by maximum likelihood algorithm with a gamma-distribution, the GTR model for nucleotide substitution and the JTT model for amino acid substitution[72].

Parsimony analyses were conducted using PAUP* 4.0b10.[68] with equal weights for 18S rDNA sequences and a weighted step matrix converted from the JTT matrix [73,74] for RPB1, RPB2 and their combined data set. Gaps were scored as missing. The heuristic search using the random addition of taxon option was performed with 1000 replicates to increase the chance of finding all of the most parsimonious trees. To evaluate the strength of the phylogenetic conclusions, 500 parsimony bootstrap replicates were performed using the heuristic search with the random addition of taxon option (10 times per replicate).

In order to test alternative phylogenetic hypotheses, analyses were conducted using PAUP to construct the most parsimonious trees under the constrained conditions, and the resulting trees were evaluated together with the most parsimonious tree and the tree of Bayesian inference in TREE-PUZZLE 5.2 using the SH test based on the combined data set of RPB1 and RPB2 [72,75]. TREE-PUZZLE 5.2 incorporates the JTT matrix for amino acid substitutions and gamma-distributed rates to allow for rate heterogeneity among sites. The gamma distribution parameter α of 0.33 was estimated from our combined dataset of RPB1 and RPB2. Eight rate categories were used in the analyses.

## Abbreviations

RPB1: the largest subunit of nuclear DNA-dependent RNA polymerase II

RPB2: the second largest subunit of nuclear DNA-dependent RNA polymerase II

rRNA: ribosomal RNA

rDNA: ribosomal DNA

EF-1α: translation elongation factor 1α

## Authors' contributions

YJL carried out the design of this study, the acquisition of data, the alignment of sequences, and performed the phylogenetic and statistical analyses. YJL also interpreted the data, drafted and revised the manuscript. MCH participated in the molecular cloning and sequencing and annotated intron positions for certain genes. BDH participated in its design and was involved in writing the manuscript. All authors read and approved the final manuscript.

## Supplementary Material

Additional File 1 **Supplementary Table**. Specimens used in this study and the GenBank accession numbers for their RPB1, *RPB*2 and 18S rRNA gene sequences. Click here for file
