# Supplementary material for: Loss of the flagellum happened only once in the fungal lineage: phylogenetic structure of Kingdom Fungi inferred from RNA polymerase II subunit genes
Source: BMC Evol Biol. 2006 Sep 29;6:74. doi: 10.1186/1471-2148-6-74 (PMC1599754; doi:10.1186/1471-2148-6-74)
Supplement: Additional File 1 — Supplementary Table. Specimens used in this study and the GenBank accession numbers for their RPB1, RPB2 and 18S rRNA gene sequences. [file 1471-2148-6-74-S1.doc]

Supplementary Table. Specimens in this study and the GenBank accession numbers for their 18S rRNA, *RPB1* and *RPB*2 gene sequences.

|  | classification ^a^ | | GenBank accession number (reference) | | |
| --- | --- | --- | --- | --- | --- |
| Speicies | Phylum | Order | 18S rDNA | RPB1 | RPB2 |
| Gibberella zeae | Ascomycota | Hypocreales | NA | XP_381092 | XP_381019 |
| Gibberella moniliformis | Ascomycota | Hypocreales | NA | NA | AY533830 (Liu and Hall 2004) |
| Magnaporthe grisea | Ascomycota | [Sordariomycetes incertae sedis](http://www.ncbi.nlm.nih.gov/Taxonomy/Browser/wwwtax.cgi?mode=Info&id=147551&lvl=3&lin=f&keep=1&srchmode=1&unlock) | AB026819 | XP_362207 | XP_362269 |
| Cryphonectria parasitica | Ascomycota | Diaporthales | NA | NA | AY485619 (Liu and Hall 2004) |
| Neurospora crassa | Ascomycota | Sordariales | X04971 | XP_329293 | XP_329740 |
| Chaetomium elatum | Ascomycota | Sordariales | M83257 | NA | NA |
| Microascus cirrosus | Ascomycota | Microascales | M89994 | NA | NA |
| Ceratocystis fimbriata | Ascomycota | Microascales | U43777 | NA | NA |
| Sclerotinia sclerotiorum | Ascomycota | Helotiales | L37541 | EF014371 (this study) | AF107808 (Liu et al. 1999) |
| Botrytis cinerea | Ascomycota | Helotiales | AY544695 | Syngenta genome sequences | AY495590 (Liu and Hall 2004) |
| Leotia viscosa | Ascomycota | Helotiales | AF113715 | NA | AF107807 (Liu et al. 1999) |
| Trichophyton rubrum | Ascomycota | Onygenales | Z34928 | EF014372 (this study) | AF107795 (Liu et al. 1999) |
| Coccidioides immitis | Ascomycota | Onygenales | M55627 | EAS32220 | EAS29067 |
| Coccidioides posadasii | Ascomycota | Onygenales | NA | TIGR 222929 | NA |
| Ajellomyces dermatitidis | Ascomycota | Onygenales | AF320010 | NA | NA |
| Ajellomyces capsulatus | Ascomycota | Onygenales | X58572 | NA | NA |
| Aspergillus fumigatus | Ascomycota | Eurotiales | M55626 | XP_752837 | AY485610 (Liu and Hall 2004) |
| Aspergillus oryzae | Ascomycota | Eurotiales | NA | BAB12227 | NA |
| Aspergillus nidulans | Ascomycota | Eurotiales | U77377 | XP_404946 | XP_413257 |
| Aspergillus terreus | Ascomycota | Eurotiales | AB008409 | NA | NA |
| Eurotium rubrum | Ascomycota | Eurotiales | U00970 | NA | NA |
| Eurotium herbariorum | Ascomycota | Eurotiales | AB008402 | NA | NA |
| Byssochlamys nivea | Ascomycota | Eurotiales | M83256 | NA | NA |
| Kluyveromyces lactis | Ascomycota | Saccharomycetales | X51830 | XP_455310 | XP_451784 |
| kluyveromyces waltii | Ascomycota | Saccharomycetales | NA | AADM01000294 | NA |
| Saccharomyces kluyveri | Ascomycota | Saccharomycetales | NA | AACE01000035 | NA |
| Saccharomyces castellii | Ascomycota | Saccharomycetales | NA | AACF01000092 | NA |
| Eremothecium gossypii | Ascomycota | Saccharomycetales | AY046265 | Q75A34 | AAS53775 |
| Saccharomyces cerevisiae | Ascomycota | Saccharomycetales | J01353 | X96876 | M15693 |
| Candida glabrata | Ascomycota | Saccharomycetales | AB094140 | XP_447415 | XP_448959 |
| Candida krusei | Ascomycota | Saccharomycetales | AB053239 | EF014373 (this study) | AF107788 (Liu et al. 1999) |
| Debaryomyces hansenii | Ascomycota | Saccharomycetales | AB054270 | XP_456921 | AAT12540 |
| Candida albicans | Ascomycota | Saccharomycetales | X53497 | EAL00529 | EAK99513 |
| Candida tropicalis | Ascomycota | Saccharomycetales | M55527 | NA | AY485615 (Liu and Hall 2004) |
| Candida parapsilopsis | Ascomycota | Saccharomycetales | NA | NA | AY485614 (Liu and Hall 2004) |
| Pichia (Candida) guilliermondii | Ascomycota | Saccharomycetales | AB105434 | NA | AY485613 (Liu and Hall 2004) |
| Yarrowia lipolytica | Ascomycota | Saccharomycetales | AB018158 | XP_501909 | XM_502376 |
| Taphrina deformans | Ascomycota | Taphrinales | U00971 | EF014374(this study) | AY485633 (Liu and Hall 2004) |
| Schizosaccharomyces pombe | Ascomycota | Schizosaccharomycetales | X54866 | NP_595673 | D13337 |
| Schizosaccharomyces japonicus | Ascomycota | Schizosaccharomycetales | AB243296 | NA | NA |
| Neolecta vitellina | Ascomycota | Neolectales | Z27393 | EF014375(this study) | AF107786 (Liu et al. 1999) |
| Pneumocystis carinii | Ascomycota | Pneumocystidales | X12708 | NA | AY485631 (Liu and Hall 2004) |
| Amanita phalloides | Basidiomycota | Agaricales | NA | AY485639 (this study) | AY485609 (Liu and Hall 2004) |
| Coprinopsis cinerea | Basidiomycota | Agaricales | M92911 | www.broad.mit.edu^b^ | www.broad.mit.edu^b^ |
| Agaricus bisporus | Basidiomycota | Agaricales | U23724 | NA | AF107785 (Liu et al. 1999) |
| Phanerochaete chrysosporium | Basidiomycota | Aphyllophorales | U59084 | AADS01000177 | http://genome.jgi-psf.org/whiterot1/whiterot1.home.html |
| Hydnum repandum | Basidiomycota | Cantharellales | AF026641 | EF014376(this study) | AY485624 (Liu and Hall 2004) |
| Cryptococcus neoformans | Basidiomycota | Tremellales | M55625 | XM_570943 | XM_570204 |
| Cryptococcus grubii | Basidiomycota | Tremellales | NA | AACO01000043 | NA |
| Phragmidium sp | Basidiomycota | Uredinales | EF014363 (This study) | EF014377(this study) | AY485630 (Liu and Hall 2004) |
| Ustilago maydis | Basidiomycota | Ustilaginales | X62396 | EAK84769 | EAK83484 |
| Smittium culisetae | Zygomycota | Harpellales | AF007540 | EF014378(this study) | EF014395(this study) |
| Capniomyces stellatus | Zygomycota | Harpellales | AF007531 | EF014379(this study) | EF014396(this study) |
| Furculomyces boomerangus | Zygomycota | Harpellales | AF007535 | EF014380(this study) | EF014397(this study) |
| Mucor hiemalis | Zygomycota | [Mucorales](http://www.ncbi.nlm.nih.gov/Taxonomy/Browser/wwwtax.cgi?mode=Info&id=4827&lvl=3&p=mapview&p=has_linkout&p=blast_url&p=genome_blast&lin=f&keep=1&srchmode=1&unlock) | AF113428 | EF014381(this study) | EF014398(this study) |
| Rhizopus oryzae | Zygomycota | Mucorales | NA | www.broad.mit.edu^b^ | www.broad.mit.edu^b^ |
| Basidiobolus ranarum | Zygomycota | Entomophthorales | D29946 | EF014382(this study) | EF014399(this study) |
| Scutellospora reticulata | Zygomycota | Diversisporales | AJ871272 | NA | NA |
| Glomus mosseae | Zygomycota | Glomerales | AY635833 | EF014383(this study) | EF014400, EF014401 (this study) |
| Gigaspora gigantea | Zygomycota | Diversisporales | EF014362 (This study) | EF014384(this study) | EF014402(this study) |
| Gigaspora rosea | Zygomycota | Diversisporales | X58726 | NA | NA |
| Gigaspora margarita | Zygomycota | Diversisporales | X58726 | NA | NA |
| Allomyces macrogynus | Chytridiomycota | Blastocladiales | EF014364 (this study) | EF014385(this study) | EF014403, EF014404(this study) |
| Catenaria anguillulae | Chytridiomycota | Blastocladiales | EF014365 (this study) | EF014386(this study) | EF014405(this study) |
| Blastocladiella emersonii | Chytridiomycota | Blastocladiales | EF014366 (this study) | EF014387(this study) | NA |
| Coelomomyces stegomyiae | Chytridiomycota | Blastocladiales | AF322406 | EF014388(this study) | EF014406(this study) |
| Entophlyctis confervae-glomeratae | Chytridiomycota | Chytridiales | EF014367 (this study) | EF014389(this study) | EF014407(this study) |
| Chytriomyces hyalinus | Chytridiomycota | Chytridiales | EF014368 (this study) | EF014390, EF014391 (this study) | EF014408, EF014409(this study) |
| Monoblepharis macrandra | Chytridiomycota | Monoblepharidales | EF014369 (this study) | AF315822 | EF014410(this study) |
| Neocallimastix frontalis | Chytridiomycota | Neocallimastigales | EF014370 (this study) | EF014394(this study) | EF014411, EF014412(this study) |
| Encephalitozoon cuniculi |  | Microsporidia | NA | CAD26175 | CAD25744 |
| Vairimorpha necatrix |  | Microsporidia | NA | AAD12604 | NA |
| Nosema tyriae |  | Microsporidia | NA | AJ278948 | NA |
| Cystosporogenes operophterae |  | Microsporidia | NA | CAC33855 | NA |
| Homo sapiens |  |  | K03432 | NP_000928 | NP_000929 |
| Mus musculus |  |  | X00686 | NP_033115 | NP_722493 |
| Rattus norvegicus |  |  | M11188 | NA | NA |
| Drosophila melanogaster |  |  | NA | NP_511124 | NP_476706 |
| Anopheles gambiae |  |  | NA | EAA12308 | EAA08858 |
| Caenorhabditis elegans |  |  | AY268117 | NP_500523 | NP_498047 |
| Monosiga ovata |  |  | AF271999 | NA | NA |
| Monosiga brevicollis |  |  | NA | AF315821 | EF014413 (this study) |
| Oryza sativa |  |  | NA | XP_493925 | XP_480298 |
| Zea mays |  |  | NA | AAQ08515 | NA |
| Arabidopsis thaliana |  |  | NC_003071 | CAA36735 | Z19121 |
| Lycopersicon esculentum |  |  | NA | DQ020644 | DQ020639 |
| Amborella trichopoda |  |  | NA | AAQ08518 | NA |
| Zamia muricata |  |  | NA | AAQ08511 | NA |
| Pinus nigra |  |  | NA | AAQ08513 | NA |
| Psilotum nudum |  |  | NA | AAQ08519 | NA |
| Spirogyra sp |  |  | NA | U90210 | NA |
| Chlamydomonas reinhardtii |  |  | AY665726 | C 1140045 | DQ020659 |
| Glaucocystis nostochinearum |  |  | NA | DQ202658 | NA |
| Botryocladia uvarioides |  |  | NA | AF315819 | NA |
| Glaucosphaera vacuolata |  |  | NA | AF315820 | NA |
| Porphyra yezoensis |  |  | NA | U90208 | NA |

## ^a^ The classification used in this study is followed Alexopoulos et al (1996).

**^b^**  http://www.broad.mit.edu/annotation/fungi/
